# Shared Concerns and Opportunity for Joint Action in Creating a Food Environment That Supports Health

**DOI:** 10.3390/nu11010041

**Published:** 2018-12-25

**Authors:** Kendrin R. Sonneville, Rachel F. Rodgers

**Affiliations:** 1 Department of Nutritional Sciences, University of Michigan School of Public Health, Ann Arbor, MI 48109, USA; 2 Department of Applied Psychology, Bouve College of Health Sciences, Northeastern University, Boston, MA 02115, USA; R.Rodgers@northeastern.edu

**Keywords:** eating disorders, obesity, prevention, food industry, food environment, food policy

## Abstract

The food industry is a for-profit industry with high relevance to universal eating disorders prevention. To date, policy which targets the food industry and food environment has been underutilized in efforts to decrease the incidence of eating disorders and associated risk factors. In contrast, food policy has been extensively leveraged with the aim of reducing the incidence of obesity. While philosophical misalignments with these later efforts may have constituted an obstacle to identifying the food environment as a key target for eating disorders prevention, food policy is an area where shared interests can be found. Specifically, a shared goal of obesity and eating disorders prevention efforts is creating a food environment that supports health, while minimizing the influence of the food industry that profits from the sale of highly palatable, processed foods and “diet” foods and from increasing portions of foods served and eaten.

## 1. Introduction 

Eating disorders are a significant public health issue and affect people of all backgrounds [1,2,3,4]. Approximately 5% of individuals in the U.S. will have an eating disorder at some point in their lives [2] and many more will struggle with sub-threshold symptoms [5,6,7], putting them at risk for substantial psychiatric comorbidities [8] and serious medical complications [9]. As evidence emerges for sociocultural drivers of eating disorder risk, recognition of the need to identify population-level approaches to eating disorders prevention has increased [10]. For-profit industries that benefit when people have an unhealthy relationship with food and their bodies (e.g., the diet, fashion, and beauty industries) have been implicated as important targets for macro-level intervention for eating disorders prevention [11,12]. The food industry, which includes food and beverage companies and quick-serve restaurants, is another such for-profit industry with high relevance to universal eating disorders prevention. To date, however, it has been neglected in efforts to decrease the incidence of eating disorders and associated risk factors [13]. In contrast, policy related to the food industry has been leveraged with the aim of reducing the incidence of obesity [14,15,16]. In this way, food policy is an area where shared interests can be found with the goal of creating a food environment that supports health. While food policy encompasses a heterogeneous set of policies involving many sectors that impact how food is produced, processed, distributed, marketed, purchased, and consumed [17], we will specifically focus on policy which targets practices of the food industry related to the processing, distribution, and marketing of foods. The aim here is to outline the rationale for leveraging food policy as an approach to eating disorders prevention. We will examine ways in which this lens would benefit ongoing efforts to regulate the food environment that largely aim to reduce individuals’ intake and will reflect upon the challenges to finding common ground in working towards informing food policy. 

## 2. The Relevance of the Food Industry to Universal Eating Disorders Prevention Efforts 

**Figure 1:**
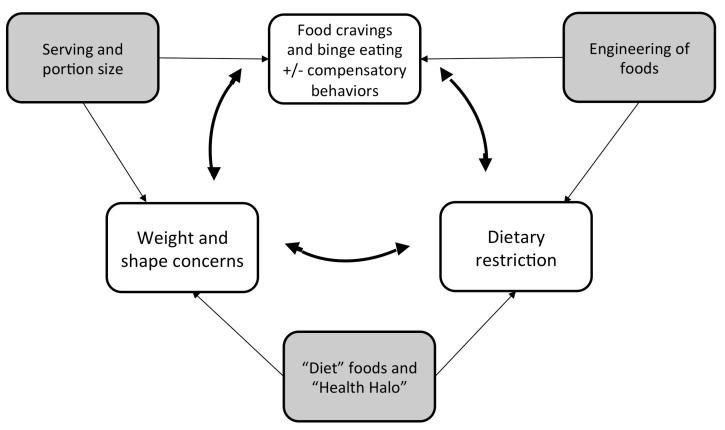
Food industry practices and eating disorder risk.

Eating disorders are characterized by an intense preoccupation with weight and shape, engagement in behaviors that aim to alter weight and shape, and/or the experience of loss of control while eating. In particular, restrictive-type eating disorders include efforts to eat less often, consume smaller quantities, and choose foods that are lower in caloric density [18]. These efforts frequently result in strict food rules and certain foods being thought of as permitted, while others are forbidden. In addition, binge-type eating disorders are characterized by the consumption of large amounts of food in a short amount of time, with an accompanying experience of loss of control [19]. These episodes may be followed by compensatory behaviors, including self-induced vomiting, fasting, over-exercise, or the use or diuretics and laxatives in an effort to offset or eliminate the calories consumed. Individuals who experience binge-eating behaviors have reported experiencing strong cravings for certain foods that may precipitate the eating episode [20]. Furthermore, avoidance of certain foods (i.e., restriction) has been shown to increase the risk for binge-eating behaviors [21]. We will discuss how practices, including the processing, distribution, and marketing of foods, employed by the food industry may be related to eating disorder symptoms, such as preoccupation with weight and shape, dietary restriction, binge-eating behaviors, and cravings. These practices include the ways in which products are developed, (“engineering” of foods), packaged (serving size and portion), and marketed (“diet” foods and “health halo”) (Figure 1). This suggests that it is important to consider the food environment as a context that could increase the risk for eating disorders.

## 3. “*Engineering*” of Foods

In recent decades, the food industry has focused its resources on developing highly palatable, processed foods that are engineered to be as rewarding as possible to their target audience by combining levels of sugar, fat, and salt [22,23,24]. This has included research on the non-linear relationships between sweetness and the appeal of a food to identify the optimal sweetness for different foods. In particular, foods marketed specifically to children, such as children’s breakfast foods, have been developed on the basis of their preference for markedly sweeter foods [25]. In addition, the food industry has paid increasing attention to developing foods that minimize satiety, such as liquids that possess a lower satiating capacity compared to solid foods [26]. The result of these practices is a range of foods that have been perfected in their capacity not only to appeal but also to fail to be satiating, so as to maximize consumption.

The engineering of foods in this manner is relevant to eating disorders prevention efforts in several ways and emerging data confirm the association between the consumption of these foods and eating disorder risk. Firstly, foods that are “engineered in ways that appear to surpass the rewarding properties of traditional foods” [24] may disturb the processes of appetite regulation. [27] Consistent with this, highly-processed foods, containing refined sugars and added fats, have been identified as being most associated with loss of control eating [28,29,30]. Furthermore, evidence from animal models of binge eating has supported the role of highly palatable, processed foods in triggering binge eating [31,32,33,34]. Relatedly, the capacity for these foods to disrupt the development of self-regulated eating processes in children has also been questioned. The role of reward in driving these eating behaviors has led researchers to draw parallels between the effects of these foods and those of addictive substances [35,36,37].

## 4. Serving and Portion Size

The contemporary Western food environment, through practices related to the presentation, packaging, and selling of food, has been linked to increasingly large serving and portion sizes of foods (e.g., [38,39]). Examples of these practices include normalizing large portion sizes through servings in restaurants, pricing scales designed to sell larger portions, displaying foods in stores in ways that increase purchasing, and selling foods in portions or packaging that exceed a typical serving size [40,41]. These practices could increase consumption [42,43,44] and thus potentiate profits, which may explain their perpetuation. Larger serving and portion sizes could also theoretically increase the risk for binge eating. Consistent with this, preliminary evidence supports the fact that the effects of large portion sizes on increased consumption are strongest among youth who experience loss of control eating or binge eating [45,46]. In addition, a food-rich environment, in conjunction with social appearance ideals of thinness/leanness and the stigmatization of larger bodies, may place some individuals under increased pressure to restrict their intake [47]. Thus, large serving and portion sizes may be associated with either binge-type eating pathology, or restriction, through different pathways and thus increase risk for eating disorders. 

## 5. The Creation of “Diet” Foods and the “Health Halo”

The rising preoccupation with weight control and discourse identifying individual eating behaviors as determinants of health has been accompanied by the creation of numerous “diet” foods (e.g., reduced-calorie or “light” food products). These products have been developed to contain low-calorie artificial sweeteners in place of sugar, reduced levels of fat, or other reformulations of nutrients. For example, the food industry profited from dietary guidelines that emphasized reducing total fat in the diet by creating numerous low-fat, high-carbohydrate, high-sugar products [48,49]. In addition, the food industry has been described as manipulating perceptions of the health benefits of their products through the use of various claims about foods [50]. Importantly, while these claims may assist individuals in their food choices, they may also be misleading [51]. 

The increasing availability and marketing of “diet” foods may be relevant to eating disorders through a number of pathways. These products may be marketed as assisting in weight control and therefore attractive to individuals with weight concerns. Meta-analytic findings have confirmed that health-related claims on foods have a substantial effect on food choices and increased purchasing [50,52], which may particularly affect those with weight and shape concerns. However, consumption of “diet” or “health” products is unlikely to alleviate weight and shape concerns, or be helpful in terms of establishing eating patterns that are regulated by internal cues. Instead, consuming artificially-sweetened beverages rather than sugar-sweetened ones has been shown to be associated with increased levels of restrained and emotional eating [53,54]. Furthermore, consuming these products may increase cravings and loss of control eating through the process of cognitive restraint [55]. In addition, “diet’ and “health” foods might substitute consumption of less processed foods, that are more helpful to appetite regulation [28]. Finally, at the macro-level, the proliferation of such foods and their intense marketing serves to increase weight-related pressures and contributes to the sociocultural discourse that promotes disordered eating [56]. In summary, the food environment may increase risk for eating disorders via multiple pathways and is therefore an important target for universal prevention efforts, particularly through policy and regulation.

## 6. Food Policy for the Prevention of Obesity

Efforts to leverage policy for the prevention of obesity are widespread and largely aim to reduce individuals’ overall intake. Public policy strategies related to obesity prevention in the U.S. and abroad have focused on a wide range of environments, including those related to child care, health care, and schools, [14] and are designed to increase the availability, affordability, and acceptability of healthy food and beverage choices in various settings (e.g., schools, workplace, community), while decreasing the availability of less healthy options in these settings [15]. While many of these policy efforts could have relevance to eating disorders and their prevention, these potential intersections have been underexplored. 

Policy efforts aimed at limiting portion sizes are an example of a food policy for obesity prevention that also has relevance for eating disorders prevention. Based on the body of research linking portion size to greater consumption [57], a number of initiatives have been undertaken, ranging from voluntary pledges to regulations [58]. In the U.S., the Food and Drug Administration (USDA) already requires a statement regarding portion sizes on the labels of foods. Furthermore, the USDA is currently in the process of implementing changes to the description of portion sizes to better reflect the amount generally consumed in the nutritional information included on the labels. These changes intend to ensure that food package labels provide a more easily interpretable estimation of the energy and nutrition provided by foods. However, because portion size signals a norm about what should be consumed, these changes could ultimately influence food consumption [43,59,60]. How such practices and the resultant policies affect eating disorders and associated risk factors warrants further investigation. 

An additional example within the U.S. is regulation that requires calorie labeling in restaurants in an effort to guide consumers towards lower-calorie options [61]. While such policies are viewed positively for increasing food industry transparency, the impact of such policies on calorie consumption has been limited [62,63,64]. Moreover, a number of concerns have been expressed regarding the ways in which such labeling might impact individuals with eating concerns, who experience high levels of preoccupation around calorie content. Indeed, recent data suggest that individuals with disordered eating behaviors [65] and those with eating disorders [66] may be particularly vulnerable to unintended negative effects of calorie labels on menus. Given the limited efficacy of these policies and their potential for unintended negative effects, greater involvement from those with expertise in eating disorders prevention during the development of such policies is warranted.

A final example of a food policy related to obesity prevention involves the levying of sugar-taxes and the regulation of serving sizes, particularly in the context of sugar-sweetened beverages [67]. It has been suggested that the increase of sugar-related taxes would have a significant positive public health impact, as well as a number of economic benefits [68]. Similarly, unsuccessful efforts made by the New York City Board of Health to regulate the size of sugar-sweetened beverages sold in that state were considered to have a strong potential for positive impact on public health [69]. However, concern regarding other unintended harmful effects, such as increases in alcohol consumption and the disproportionate impact on low-income groups, have been voiced [70]. Furthermore, in the context of eating disorders, such policies may contribute to maintaining the focus on weight as opposed to health and thus perpetuate appearance-related pressures [56]. Thus, again, the inclusion of eating disorders prevention perspectives in the development of such policies is critical.

Broadly, efforts to regulate the food environment that have aimed to prevent obesity have targeted aspects of the food environment that are of relevance to the prevention of eating disorders, namely, the lack of transparency of the industry with regard to the nutritional content of foods, as well as the selling and marketing practices that aim to increase profit. As described above, these practices are those through which the food environment may increase risk for eating disorders. Therefore, policies that are capable of altering these practices could serve eating disorders prevention efforts.

## 7. Challenges to Integrating Eating Disorders and Obesity Prevention Policy 

Common to obesity prevention and eating disorders prevention efforts is the desire to improve public health and reduce factors that drive disparities in health. As such, a shared goal of these prevention efforts would include access to a food environment that promotes health, while minimizing the influence of the food industry that profits from the sale of highly palatable, processed foods and “diet” foods, and from increasing portions sizes. Several challenges to targeting the food industry exist that relate to philosophical misalignments between the perspectives of eating disorders and obesity prevention efforts, however. 

The first challenge relates to the extent to which weight is a primary indicator of health and as the primary rationale for changing food policy. Obesity prevention efforts are designed to reduce the prevalence of obesity using a definition based on body mass index (BMI). Using this framing to justify policy changes implies that weight gain is inherently unhealthy and crudely categorizes smaller bodies as “healthy” and larger bodies and “unhealthy” or “diseased.” Weight stigma scholars and fat advocates generally reject the term obesity for this reason—that it pathologizes fat bodies, ignores natural size diversity, and, by relying solely on BMI, fails to measure health more holistically [71,72]. Within eating disorders prevention, experts generally reject the idea that weight gain is unhealthy (indeed, weight gain is a necessary and healthy part of recovery for many people with eating disorders, irrespective of weight), yet the erratic and disordered eating habits and/or binge eating behavior that may be associated with weight gain should be considered problematic. To the extent that the food industry contributes to and profits from this type of eating, eating disorders prevention must target their practices, while also advocating for weight-neutral approaches and advocating against weight bias.

A second challenge relates to the types of policy changes that are proposed. Many obesity prevention policy efforts focus on specific foods (e.g., sugar sweetened beverages, fast food, etc.). This approach is seemingly incompatible with evidence-based eating disorders frameworks based on moderation and trusting one’s own internal cues of hunger, taste, and fullness (e.g., Health at Every Size^®^, intuitive eating, mindful eating, etc.) [73,74,75]. Many individuals with eating disorders assign “good” or ”bad” labels to foods or have “forbidden foods” that they avoid eating out of fear or guilt. Learning to eat these in moderation has been shown to be a key element of successful treatment [76]. As such, eating disorders prevention messaging often adopts a “no good/bad foods” mantra. This perspective represents a substantial barrier to joining efforts to limit access to or penalize certain foods. Importantly, the food environment has changed considerably in the last few decades. Accordingly, a more nuanced perspective on food quality may better reflect the current reality of the food environment, and may need to be incorporated into approaches for the prevention of eating disorders. Individuals who are at risk for developing eating disorders may be particularly vulnerable to the presence in the food environment of foods that are designed to appeal to hedonic drives and override homeostatic drives. As such, the prevention of eating disorders includes the criticism of tactics used by the food industry that may capitalize on this vulnerability.

Philosophical differences and key concerns with messaging underlying obesity prevention policies do present an obstacle to integrating eating disorders prevention perspectives into existing efforts that target the food industry. These differences have contributed to the absence of efforts to leverage food policy as a means of the universal prevention of eating disorders. By steering clear of these efforts, however, the considerations that are unique and critical to efforts to prevent eating disorders are left out of the larger conversation related to the regulation of the food environment. Thus, the food industry benefits from minimal oversight related to their contribution to eating disorder risk.

## 8. Conclusions

The food industry is a key player in the creation of a food environment, characterized by the over-abundance and aggressive marketing of engineered foods that may increase risk for eating disorders or negatively impact their remission. Developing and implementing effective policy approaches to limit the food industry’s capacity to continue shaping the food environment in this way is emerging as a critical goal for universal prevention efforts. However, the capacity for these efforts to pathologize larger bodies or vilify specific foods has constituted an obstacle to considering how the broader goal of obesity prevention efforts (i.e., creating a food environment that supports health) may also serve eating disorders prevention. An unintended consequence of this position may be the proliferation of food industry practices that might increase eating disorder risk. Meanwhile, those who are highly susceptible to the presence of these foods or food industry practices may suffer. Eating disorders experts have a crucial part to play in informing policies targeting the food industry to ensure that such policies do no harm. Furthermore, policy efforts targeting the food industry should be considered as a means of improving universal prevention and decreasing an important environmental risk for eating disorders.
